# Artemisinin Alleviates Intestinal Inflammation and Metabolic Disturbance in Ulcerative Colitis Rats Induced by DSS

**DOI:** 10.1155/2022/6211215

**Published:** 2022-04-19

**Authors:** Xuemei Jia, Yunxiao Gao, Liran Liu, Yuxi Guo, Jie Wang, Hongyu Ma, Runyuan Zhao, Bolin Li, Yao Du, Qian Yang

**Affiliations:** ^1^Hebei University of Chinese Medicine, Shijiazhuang, Hebei 050091, China; ^2^Department of Traditional Chinese Medicine, Hebei General Hospital, Shijiazhuang, Hebei 050051, China; ^3^Department of Gastroenterology, The First Affiliated Hospital of Guangzhou University of Chinese Medicine, Guangdong, Guangzhou 510405, China; ^4^Department of Gastroenterology, The First Affiliated Hospital of Hebei University of Chinese Medicine, Shijiazhuang, Hebei 050011, China

## Abstract

**Objective:**

This study is aimed to reveal the possible mechanisms of artemisinin in the treatment of ulcerative colitis (UC) through bioinformatics analysis and experimental verification in UC model rats.

**Methods:**

Firstly, we searched two microarray data of the Gene Expression Omnibus (GEO) database to explore the diﬀerentially expressed genes (DEGs) between UC samples and normal samples. Then, we selected DEGs for gene ontology (GO) function enrichment analysis and Kyoto Encyclopedia of Genes and Genomes (KEGG) pathway enrichment analysis. The acute UC model of rats was established by using 3.5% dextran sulfate sodium (DSS) for 10 days to verify the core pathway. Finally, we evaluated the therapeutic effect of artemisinin at the molecular level and used metabonomics to study the endogenous metabolites in the rat serum.

**Results:**

We screened in the GEO database and selected two eligible microarray datasets, GSE36807 and GSE9452. We performed GO function and KEGG pathway enrichment analyses of DEGs and found that these DEGs were mainly enriched in the inflammatory response, immune response, and IL-17 and NF-*κ*B signaling pathways. Finally, we verified the IL-17 signaling pathway and key cytokines, and ELISA and immunohistochemical results showed that artemisinin could downregulate the expression of proinflammatory cytokines such as IL-1*β* and IL-17 in the IL-17 signaling pathway and upregulate the expression of the anti-inflammatory cytokine PPAR-*γ*. Metabolomics analysis showed that 33 differential metabolites were identified in the artemisinin group (AG) compared to the model group (MG). Differential metabolites were mainly involved in alanine, aspartate, and glutamate metabolism and synthesis and degradation of ketone bodies.

**Conclusion:**

In this study, we found that artemisinin can significantly inhibit the inflammatory response in UC rats and regulate metabolites and related metabolic pathways. This study provides a foundation for further research on the mechanism of artemisinin in the treatment of UC.

## 1. Introduction

Ulcerative colitis (UC) is a chronic nonspecific intestinal inflammation characterized by abdominal pain, diarrhea, and blood in the stool [1]. The pathogenesis of UC is not completely clarified and may be related to immune, environmental, and genetic factors. The anti-UC drugs mainly include 5-aminosalicylic acid, hormones, immunosuppressive agents, etc. But UC cannot be cured completely and greater side effects were left in UC patients [2]. With the development of industrialization and urbanization, the number of diagnosed UC patients in China has increased sharply, with 1.2 cases per 100,000 people, and this number is still increasing [3]. About half of UC is classified as a chronic recurrent disease [4]. The long course and recurrent episodes often afflict the mental and psychological state of patients, and the prolonged course also leads to an increased risk of colorectal cancer [5].

The exact pathogenesis of UC is unknown. Studies have shown that bioinformatics technology can help us understand the pathogenesis of UC more clearly by identifying different genes in UC microarray data [6, 7].

Compared with chemical drugs, traditional Chinese medicines have the advantage of fewer side effects, and currently attract researchers' attention to the treatment of gastrointestinal diseases [8]. Artemisinin is a semiterpene lactone compound, which has been listed by the World Health Organization as a first-line drug for the treatment of malaria [9]. In addition to antimalarial, artemisinin also has anti-inflammatory, antibacterial, antitumor, antifibrosis, and immune regulation effects [10]. Studies have found that artemisinin can treat immune diseases by inducing the production of regulatory T cells and inhibiting the phosphorylation of AKT [11], and it can play an anti-inflammatory role by inhibiting the activation of NLRP3 inflammasome [12]. According to reports, artemisinin can ameliorate inflammation-driven lymphangiogenesis via VEGF-C/VEGFR-3 signaling pathway [13], and it can also induce the expression of CYP3A by activating PXR [14], thereby preventing inflammatory bowel disease. The treatment of artemisinin in immune and inflammatory diseases inspired us to explore the treatment of ulcerative colitis.

In recent years, metabolomics is widely used to discover the pathogenesis of diseases and the potential mechanism of action of drugs [15]. Metabolomics is a discipline that simultaneously qualitatively and quantitatively analyzes all low-molecular-weight metabolites of a certain organism or cell in a specific physiological period, and it can more accurately reflect the state of the biological system of small molecule metabolites in the treatment process from the overall level, which conforms to the holistic, dynamic, and dialectical views of Chinese medicine [16–18]. It has been confirmed that UC will cause metabolic disorders [19]. However, there are few studies on the metabolic changes of artemisinin in the treatment of UC. Therefore, it is meaningful to study whether artemisinin can reduce colon inflammation by reversing metabolism.

In this study, we applied two datasets from the Gene Expression Omnibus (GEO) database to identify the diﬀerentially expressed genes (DEGs) of UC and performed gene ontology (GO) functional and Kyoto Encyclopedia of Genes and Genomes (KEGG) pathway enrichment analyses on DEGs. Then, we constructed an acute UC model induced by dextran sulfate sodium (DSS) and verified the core pathways. Finally, we evaluated the therapeutic effect of artemisinin at the molecular level and used metabonomics to study the endogenous metabolites in the rat serum. The therapeutic effect of artemisinin on UC is provided on a theoretical basis.

## 2. Materials and Methods

### 2.1. Bioinformatics Analysis

#### 2.1.1. Data Resource

We searched in the GEO database (http://www.ncbi.nlm.nih.gov/geo/) and collected the gene expression data of GSE36807 and GSE9452 in colon mucosal tissues from healthy individuals and UC patients. Microarray data included in GSE36807 and GSE9452 were obtained from GPL570-55999 platforms. GSE36807 included 7 healthy human colon tissue samples and 15 UC patients' colon tissue samples. GSE9452 included 5 healthy human colon tissue samples and 21 UC patients' colon tissue samples.

#### 2.1.2. Data Preprocessing

The *R* language packages “Affy” and “affyPLM*”* were applied for background correction and normalization of the raw data. Then, according to the platform annotation information, each probe ID was converted into a gene ID. Subsequently, gene IDs were converted into gene symbols and saved. The *R* software “limma” package was used to screen DEGs between patients with UC and healthy human colon tissues, and the DEGs data of the intersection between the two were visualized with online software Venn diagram.

#### 2.1.3. Gene Ontology and KEGG Pathway Enrichment Analyses

Both GO and KEGG enrichment analyses of the DEGs were performed using the *R* package clusterProfiler (version 4.0.3). The results of the analysis were expressed in the form of bar graphs and bubble graphs.

### 2.2. Experimental Verification

#### 2.2.1. Drugs and Reagents

DSS was purchased from Meilun Biotechnology Co., Ltd. (Dalian, China). Mesalazine was purchased from Losan Pharma GmbH (Freiburg im Breisgau, Germany). Artemisinin was purchased from TCI Development Co., Ltd. (Shanghai, China). ELISA kits for IL-1*β*, IL-13, IL-33, and ST2 were purchased from Jiangsu Enzyme Immune Industrial Co., Ltd. (Jiangsu, China). ELISA kit for IL-17 was purchased from Shanghai Senxiong Technology Industry Co., Ltd. (Shanghai, China). ELISA kit for IL-23 was purchased from Shanghai XiTang Biological Technology Co., Ltd., (Shanghai, China).

#### 2.2.2. Animal

A total of 40 SPF male Wistar rats (age, 8 weeks; weight, 200 ± 20g) were provided by the Animal Experiment Center of Hebei Medical University. All rats were kept in a special pathogen-free (SPF) room at a temperature of 23–25°C, in a 12-hour light and 12-hour dark cycle. They had free access to water and food and acclimatized for one week to enter the experiment. This experiment was approved by the Ethics Committee and Animal Experiment Committee of Hebei Academy of Traditional Chinese Medicine.

#### 2.2.3. Experimental Design

DSS was used to induce the acute UC model. According to the random number table method, 40 rats were divided into a normal group (NG, *n* = 10) and a control group (*n* = 30). The NG rats were given normal drinking water, and the control group rats were given 3.5% DSS solution for 10 consecutive days to establish acute UC model rats. After 10 days, 3 rats were selected randomly and sacrificed from the control group for pathological evaluation. Pathological changes such as congestion, edema, erosion, and ulcer appeared in the colon tissue, indicating that the model was successful. Three rats died during the modeling period. The successful control rats were randomly divided into three groups, model group (MG, *n* = 8), western medicine group (WG, *n* = 8), and artemisinin group (AG, *n* = 8). The WG was administered with 0.315 g/kg/d of mesalazine suspension, and AG was administered with 100 mg/kg/d of artemisinin suspension. NG and MG were given the same dose of 0.9% saline. After 14 days of treatment, the rats were fasted for 24 hours. Rats were injected intraperitoneally with 1% sodium pentobarbital (50 mg/kg).

#### 2.2.4. Disease Activity Index (DAI)

According to the DAI scoring standard established by Murano et al. [20], DAI = (weight loss score + stool trait score + stool blood score)/3 (Table 1).

#### 2.2.5. Histopathological Evaluation

For hematoxylin-eosin (HE) staining, the paraffin sections of colon tissue that have been fixed and sectioned were soaked in xylene for dewaxing, hydrated in absolute ethanol, soaked in different gradients of ethanol, fully stained with hematoxylin staining solution, rinsed with distilled water, differentiated with hydrochloric acid and ethanol, rinsed with double distilled water, fully immersed in eosin staining solution, dehydrated with different gradients of ethanol, immersed in xylene, air dried, sealed with neutral gum, and observed under a microscope.

#### 2.2.6. Immunohistochemistry

According to the immunohistochemical process, the contents of NF-*κ*B and PPAR-*γ* in colon tissue were detected. The colon tissue sections were deparaffinized and hydrated, washed three times with PBS buffer, and then boiled with 0.01 M sodium citrate buffer for antigen retrieval. Endogenous peroxidase and biotin were inactivated with 3% hydrogen peroxide. Sections were blocked with 5% BSA, rabbit antirat NF-*κ*B polyclonal antibody and rabbit antirat PPAR-*γ* polyclonal antibody were incubated overnight at 4°C, and secondary antibodies (1:500; Abcam, MA, US) at room temperature for 1 hour. Analysis was performed using the HMIAS-2000 imaging system, and NF-*κ*B and PPAR-*γ* positive areas were observed under a microscope (Olympus, Tokyo, Japan).

#### 2.2.7. Serum Sample Preparation

The anesthetized rat was placed on the operating table, of which 3–5 ml of blood was taken from the heart. Blood specimens were collected and centrifuged at 3000r/min at 4°C for 15 min. Then, separated serum samples were stored in −80°C refrigerator until testing.

#### 2.2.8. Biochemical Analysis

The levels of IL-1*β*, IL-17, IL-23, IL-13, IL-33, and ST2 were measured by ELISA kit.

#### 2.2.9. Metabolite Extraction

All serum samples were melted at 4°C, 100 *μ*L serum was accurately pipetted into a 2 mL centrifuge tube, 100 *μ*L internal standard mixture and 400 *μ*L methanol (−20°C) were added, vortexed for 1 min; centrifuged at 12000 rpm and 4°C for 10 min, and 500 *μ*L of the supernatant was placed in a 2 ml centrifuge tube and concentrated and dried in vacuo. About 150 *μ*L of 80% methanol solution was reconstituted, and after mixing, centrifuged at 12000 rpm and 4°C for 10 min, and the supernatant was taken as the sample to be tested. About 20 µL is taken from each sample to be tested and mixed into a QC sample, and the remaining samples were used for LC-MS testing.

#### 2.2.10. Liquid Chromatography-Mass Spectrometry (LC-MS) Analysis

Chromatographic separation was performed on a Thermo Vanquish system with an ACQUITY UPLC® HSS T3 (150 × 2.1 mm, 1.8 µm, Waters) column maintained at 40°C. The autosampler temperature was maintained at 8°C. The analyte was gradient eluted with 0.1% formic acid aqueous solution (A1) and 0.1% formic acid acetonitrile (B1) or 5 mM ammonium formate aqueous solution (A3) and acetonitrile (B3) at a flow rate of 0.25 mL/min. The gradient elution program was 0–1 min, 2% B1/B3; 1–9 min, 2%–50% B1/B3; 9–12 min, 50%–98% B1/B3; 12–13.5 min, 98% B1/B3; 13.5–14 min, 98%–2% B1/B3; 14–20 min, 2% B1-positive mode (14–17 min, 2% B3-negative mode).

Using the Thermo *Q* Exactive mass spectrometer, the ESI-MSn experiments were performed in positive and negative modes with the spray voltage of 3.8 kV and −2.5 kV, respectively. Sheath gas and auxiliary gas were set at 30 and 10 arbitrary units, respectively. The capillary temperature was 325°C, the full scan was carried out with a resolution of 70,000, and the scanning range was m/*z* 81–1,000. Data-dependent acquisition (DDA) MS/MS experiments were acquired with an HCD scan, and the normalized collision energy was 30 eV. Dynamic exclusion was performed to remove unnecessary MS/MS information.

#### 2.2.11. Metabolite Identification and Statistical Analysis

The obtained original data were converted to mzXML format (xcms input file format) by Proteowizard software (v3.0.8789). R's XCMS package (v3.3.2) was used for peaks identification, peaks filtration, and peaks alignment. The main parameters were bw = 2, ppm = 15, peakwidth = *c*(5, 30), mzwid = 0.015, mzdiff = 0.01, and method = centWave. The peak areas of data of different magnitudes were normalized in batches. In order to obtain more reliable and intuitive results, the data were performed unit variance scaling (UV) before multivariate statistical analysis. Principal component analysis (PCA), partial least-squares discriminant analysis (PLS-DA), and orthogonal partial least-squares discriminant analysis (OPLS-DA) were used to analyze all sample compounds. Metabolites with *p* value ≤ 0.05 ＋ VIP ≥1; *p* value ≤ 0.05 ＋ fold_change ≥1.5 or ≤0.667; one-way ANOVA *p* value ≤ 0.05 and VIP ≥1; two-way ANOVA *p* value ≤ 0.05 were considered differential metabolites. The identification of metabolites was first confirmed by the exact molecular weight of the metabolite (molecular weight error <15 ppm), then the fragment information was obtained according to the MS/MS model and annotated in the Metlin (http://metlin.scripps.edu) and MoNA (https://mona.fiehnlab.ucdavis.edu//) databases to obtain accurate metabolite information. Significantly altered metabolite data were performed using MetaboAnalyst (https://www.metaboanalyst.ca) for metabolic pathway analysis. HMDB (Human Metabolome Database, http://www. hmdh. ca) and KEGG (Kyoto Encyclopedia of Genes and Genomes, https://http://www.kegg.jp) were used to annotate differential metabolism and enrich related metabolic pathways.

## 3. Results

### 3.1. Results of Bioinformatics Analysis

#### 3.1.1. Core Differential Genes

**Figure 1:**
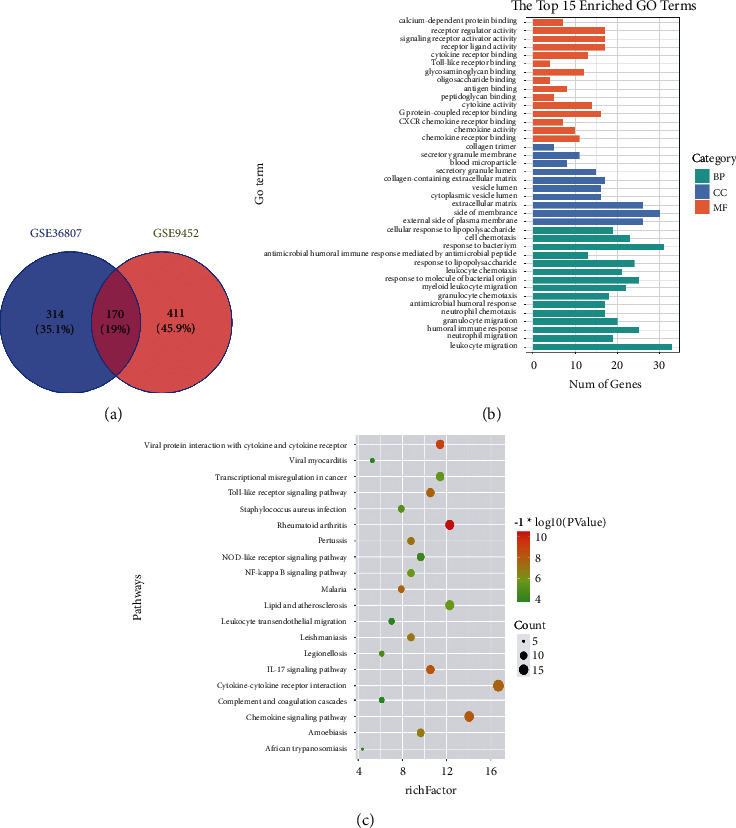
Identification of DEGs in two databases (GSE36807 and GSE9452) and GO analysis and KEGG pathway analysis of DEGs in UC. (a) Venn diagram between the common targets of two microarray data. (b) GO function analysis of intersection DEGs (top 15). (c) KEGG pathway bubble diagram of the intersection DEGs (top 20).

According to the screening conditions, the GSE36807 database contained a total of 484 DEGs, including 109 upregulated genes and 375 downregulated genes; the GSE9452 database contained 581 DEGs, including 173 upregulated genes and 408 downregulated genes. Venn software (version 2.1.0) was used to generate Venn diagrams of overlapping DEGs in 2 databases (Figure 1(a)), including 32 upregulated genes and 138 downregulated genes (Table S1).

#### 3.1.2. GO and KEGG Enrichment Analyses

GO function and KEGG pathway enrichment analyses were performed on 170 common DEGs using *R* language software. GO function enrichment analysis includes the biological process (BP), cellular component (CC), and molecular function (MF).

According to the adjustment order of *P* value from small to large, the first 15 items were selected for analysis. Among them, BP was mainly related to functions such as leukocyte migration, humoral immune response, and cell response to lipopolysaccharide; CC was mainly associated with the extracellular matrix, cytoplasmic vesicle lumen, and collagen-containing extracellular matrix; MF mainly related to the chemokine activity, cytokine activity, *G* protein-coupled receptor binding, and other functions (Figure 1(b)). A total of 32 signal pathways were obtained through KEGG enrichment analysis (adjusted *p* < 0.01). The data were screened according to the *P* value, and the first 20 entries were selected for enrichment analysis, including the IL-17 signal pathway, NF-*κ*B signal pathway, and chemokine signal pathway (Figure 1(c) and Table S2).

### 3.2. Results of Experimental Verification

#### 3.2.1. Artemisinin Can Significantly Reduce Colon Injury and Disease Activity in UC

**Figure 2:**
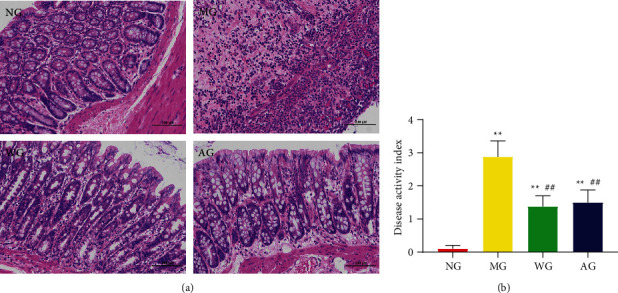
Artemisinin attenuates colonic inflammation induced by DSS. (a) HE staining (×200) of colon tissue in each group. (b) Disease activity index (DAI) in each group. NG: normal group; MG: model group; WG: western medicine group; AG: artemisinin group. ^*∗∗*^*P* < 0.05 vs NG. ^##^*P* < 0.01 vs MG.

In order to explore the effect of artemisinin on the histological changes of DSS-induced UC model rats, we used HE staining to observe the rat colon tissue. As shown in Figure 2(a), the morphology of the colon of the rats in the NG was normal, the glands were neatly arranged, the crypts were normal, and there were no inflammatory cell infiltration, congestion, and edema. In the MG, the structure of the colonic mucosa was damaged obviously, the crypt structure was disordered, the goblet cells were reduced, the neutrophils were infiltrated, and there were crypt abscesses. After the intervention of mesalazine and artemisinin, the colon mucosa of the rats was relatively complete, the glands were arranged regularly, and there were slight congestion, edema, and inflammatory cell infiltration.

Compared with the NG, the DAI score of rats in the MG increased significantly, and after the intervention of artemisinin, the DAI score decreased significantly (Figure 2(b)). The above results indicated that artemisinin can significantly reduce colon injury and disease activity in UC rats.

#### 3.2.2. Artemisinin Downregulated Proinflammatory Cytokines and Upregulated Anti-Inflammatory Cytokine in the IL-17 Signaling Pathway

**Figure 3:**
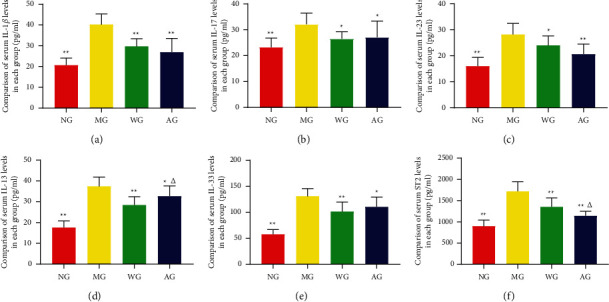
Effects of artemisinin on IL-17 signaling pathway and serum inflammatory factor expression in UC rats (n = 8 or 10). (a) IL-1*β* levels. (b) IL-17 levels. (c) IL-23 levels. (d) IL-13 levels. (e) IL-33 levels. (f) ST-2 levels. ^*∗*^*P* < 0.05. ^*∗∗*^*P* < 0.01 vs MG. ^Δ^*P* < 0.05 vs WG.

In order to verify the effect of artemisinin on inflammation in UC rats, we selected the IL-17 signaling pathway and key cytokines for detection. The ELISA results showed that compared with the NG, the levels of serum IL-1*β*, IL-17, IL-23, IL-13, IL-33, and ST2 in the MG were significantly increased (*P* < 0.05). However, artemisinin treatment significantly decreased the expression levels of them in UC rats (Figure 3).

**Figure 4:**
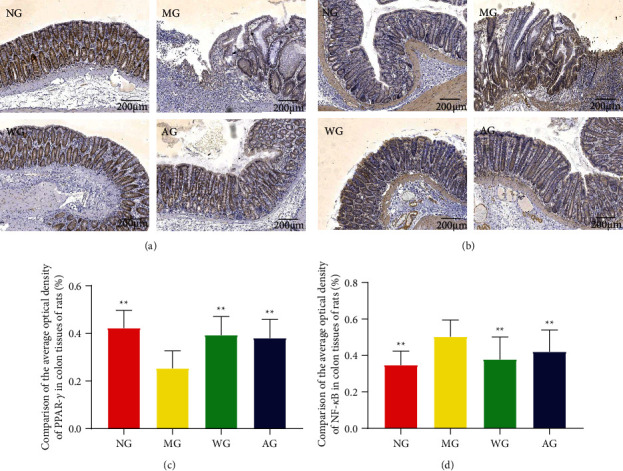
The effect of artemisinin on the expression of PPAR-*γ* (a) and NF-*κ*B (b) by immunohistochemistry (n = 8 or 10). Quantitative analyses of AOD values of PPAR-*γ* (c) and NF-*κ*B (d) at 100×magniﬁcation. ^*∗∗*^*P* < 0.01 vs MG.

The results of immunohistochemistry showed that the expression level of PPAR-*γ* in the MG was significantly downregulated than that in the NG, and artemisinin administration significantly increased the expression level of PPAR-*γ* (Figures 4(a) and 4(c)). Different from this result, the expression level of NF-*κ*B in the MG was significantly upregulated than that in the NG, and artemisinin administration significantly reduced the expression level of NF-*κ*B (Figures 4(b) and 4(d)). These results indicated that artemisinin may inhibit the colonic inflammatory response in UC rats.

#### 3.2.3. Liquid Chromatography-Mass Spectrometry (LC-MS) Method Verification

**Figure 5:**
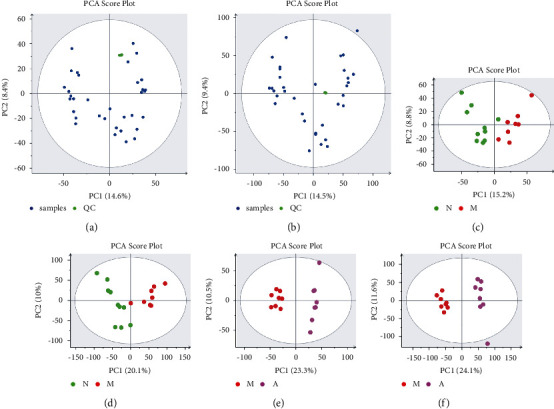
(a) PCA score plots of QC samples in positive ion mode; (b) PCA score plots of QC samples in negative ion mode; (c) PCA model results of NG and MG in positive ion mode; (d) PCA model results of NG and MG in negative ion mode; (e) PCA model results of MG and AG in positive ion mode; (f) PCA model results of MG and AG in negative ion mode.

To obtain reliable and high-quality metabolomics data, we carried out quality control (QC) and quality assurance (QA). As shown in Figures 5(a) and 5(b), the PCA score plot showed that the QC samples were clustered together, which indicated that the data were reliable and reproducible.

#### 3.2.4. Multivariate Statistical Analysis of Metabolites of Artemisinin in the Treatment of UC

In order to study the effect of artemisinin on the metabolic profile of UC rats' serum samples, we used PCA and PLS-DA for pattern recognition of the metabolite profiles in NG, MG, WG, and AG serum samples. The PCA score chart was established. In the positive and negative ion modes, the NG and the MG were significantly separated, indicating that DSS-induced UC rats had significant changes in physiology and metabolism (Figures 5(c) and 5(d)). The MG was significantly different from the AG (Figures 5(e) and 5(f)). It indicated that the overall metabolism of rats after the intervention of artemisinin was significantly different from that of the MG.

The OPLS-DA supervised pattern recognition method was employed to identify the different metabolites between each group. In the PLS-DA permutation experiment plots (Figure 5), the results of *R*^2^ and Q^2^ values generated by any random permutation on the left were lower than the origin on the right, which proved that the model had not been excessively fitting, and the results had high reliability.

**Figure 6:**
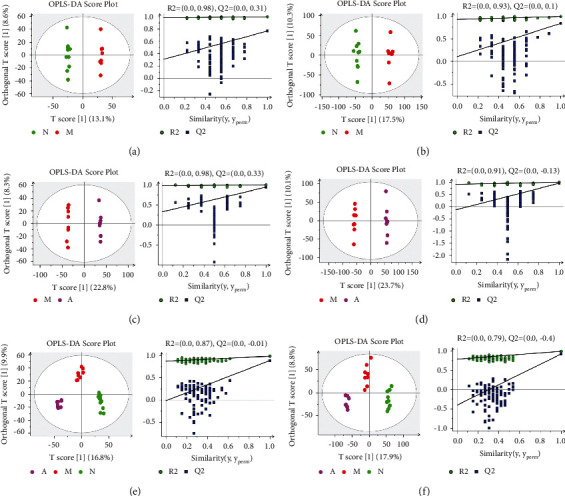
The OPLS-DA score plots and PLS-DA's 100 times permutation tests: (a) NG vs MG scores in positive ion mode; (b) NG vs MG scores in negative ion mode; (c) MG vs AG scores in positive ion mode; (d) MG vs AG scores in negative ion mode; (e) NG, MG and AG scores in positive ion mode; (f) NG, MG and AG scores in negative ion mode (OPLS-DA's model parameters: (a) R^2^Y = 0.997, *Q*^2^ = 0.733; (b) R^2^Y = 0.991, *Q*^2^ = 0.831; (c) R^2^Y = 0.999, *Q*^2^ = 0.921; (d) R^2^Y = 0.998, *Q*^2^ = 0.936; (e) R^2^Y = 0.988, *Q*^2^ = 0.909; (f) R^2^Y = 0.992, *Q*^2^ = 0.937).

The results of OPLS-DA analysis showed that the NG and the MG were significantly separated under the positive and negative ion modes, indicating that the metabolism of DSS-induced model rats had significantly changed compared with healthy rats (Figures 6(a) and 6(b)). The NG, MG, and AG were significantly separated, and the AG was located between the NG and the MG, indicating that after the treatment of artemisinin, the metabolism of DSS-induced model rats tended to improve to a normal state (Figures 6(e) and 6(f)).

#### 3.2.5. Identification of Potential Biomarkers

The VIP value and *p* value were applied to show the importance of metabolites. VIP>1 and *p* < 0.05 were used as the criteria to screen differential metabolites. Compared with the NG, there were 17 metabolites significantly increased in the MG, mainly including pyrrolidonecarboxylic acid, L-malic acid, L-carnitine, isocitric acid, indoleglycerol phosphate, dethiobiotin, gluconic acid, and phenylacetic acid, and 16 metabolites were significantly reduced, mainly including jasmonic acid, homovanillin, gentisic acid, pyrimidodiazepine, cysteine-S-sulfate, 1H-indole-3-acetamide, 5-methylcytosine, and p-aminobenzoic acid. After the intervention of artemisinin, these biomarkers showed a significant reversal (Table 2).

#### 3.2.6. Metabolic Pathway Analysis

**Figure 7:**
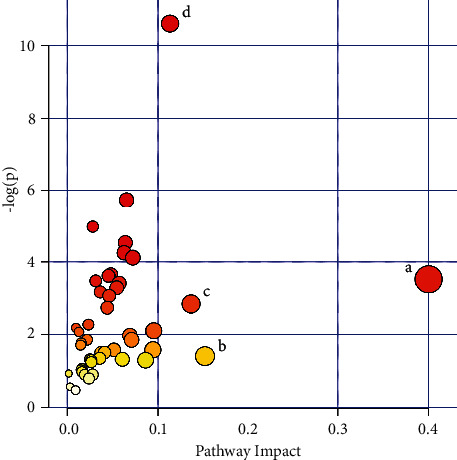
Metabolic pathways involved in potential markers in plasma. (a) Renal cell carcinoma; (b) alanine, aspartate, and glutamate metabolism; (c) synthesis and degradation of ketone bodies; (d) central carbon metabolism in cancer.

Metabolic pathways of potential biomarkers identified in UC rats were analyzed using KEGG and MetPA databases. The impact value > 0.1 was considered as a significant difference. We imported the above 33 metabolites related to the occurrence of UC into the MetPA database to explore the potential pathways of artemisinin in the treatment of UC. The results showed that artemisinin treatment of UC was mainly related to four metabolic pathways: renal cell carcinoma, alanine, aspartate, and glutamate metabolism, synthesis and degradation of ketone bodies, and central carbon metabolism in cancer (Figure 7 and Table S3). Among these pathways, alanine, aspartate, and glutamate metabolism, and synthesis and degradation of ketone bodies were related to the development of UC. The above results indicated that artemisinin changed these biomarkers and related metabolic pathways to play a therapeutic effect on UC.

## 4. Discussion

In recent years, with the development of industrialization, the incidence of UC has shown a significant upward trend in the world [21]. Although studies have shown that multiple factors such as genetics, immunity, environment, and intestinal microecology may lead to the occurrence of UC, the specific mechanism of UC is still unclear [22, 23]. Due to the complexity of the pathogenesis of UC and the lack of specific biomarkers, it greatly increases the medical burden and physical and mental discomfort of patients [24]. Bioinformatics methods can help us study and understand the underlying mechanism of UC more clearly. To find a solution to this problem, we used the GEO database to screen the datasets and verified the relevant targets.

The GEO database is an international public repository of high-throughput microarray and next-generation sequencing functional genome datasets submitted by the research community [25]. We screened in the GEO database and selected two eligible datasets, GSE36807 and GSE9452. After data analysis, we got 170 overlapping DEGs, including 32 upregulated genes and 138 downregulated genes. Then, we performed GO function and KEGG pathway enrichment analyses on the common DEGs of the two groups of microarray data and found that the pathogenesis of UC was related to inflammation and immune response, mainly enriched in IL-17 signaling pathway and NF-*κ*B signaling pathway, suggesting that inflammation and abnormal activation of the immune system may be the core features of the pathogenesis of UC [26].

Based on the current data, we believed that the IL-17 signaling pathway is more likely to reflect the degree of inflammation in DSS-induced colitis rats. Therefore, we established a UC rat model and verified the IL-17 signaling pathway and inflammation-related indicators.

The IL-17 signaling pathway is one of the important mechanisms affecting the occurrence and development of UC. NF-*κ*B plays a key pivotal role in the pathogenesis of UC, and activated NF-*κ*B can release various cytokines and immune mediators including IL-17 and activate the IL-17 signaling pathway [27]. IL-17 is a specific effector secreted by Th17 cells, which has a strong inflammatory effect. It promotes the production of IL-6, IL-1*β*, and other inflammatory factors through recruitment, mobilization, and activation of macrophages and neutrophil granulocytes and mediates inflammatory invasion and tissue damage [28]. As a proinflammatory factor, IL-23 is involved in the formation and expansion of IL-17. When the IL-23/IL-17 inflammatory axis is formed, the inflammatory response continues to expand [29]. In addition, Peter et al. found that the IL-33/ST2 axis can aggravate Dss-induced colitis and IL-33 can specifically induce the activation of key pathogenic cytokines including IL-17 and IL-13 and maintain and amplify the inflammatory response of the IL-17 signaling pathway [30]. PPAR-*γ* is a key receptor for 5-ASA and has a protective effect on mucosal damage induced by the IL-17 signaling pathway by suppressing the activation of NF-*κ*B [31, 32]. The experimental results showed that artemisinin inhibited the expression of proinflammatory cytokines such as IL-17 and IL-1*β* in the IL-17 signaling pathway and upregulated the expression of PPAR-*γ*, suggesting that artemisinin can alleviate the inflammatory immune response of UC rats.

Studies have reported that metabolomics may become an important approach for the early diagnosis of UC and provide a tool for exploring the metabolic regulation mechanism of UC. Therefore, for further exploring the therapeutic effect of artemisinin on UC, we used LC-MS to analyze the serum metabolic profiles of DSS rats and artemisinin-treated rats. PCA and PLS-DA analyses showed that 33 different metabolites could be significantly distinguished between artemisinin-treated and DSS-treated rats, mainly involving amino acid metabolism, energy metabolism, and other biochemical processes.

Amino acids play an important role in maintaining intestinal health by acting as substrates for protein synthesis in intestinal mucosal cells and modulators of metabolic pathways [33]. Amino acid metabolism mainly affects the material metabolism, energy supply, and intestinal mucosal barrier of UC [34–36] and is also closely related to the process of cell proliferation, differentiation, and apoptosis [37]. Currently, amino acid supplementation has been explored as a treatment for UC [38, 39]. This search found that DSS-induced colitis rats had significant abnormalities in amino acid metabolism, among which the contents of L-aspartic acid, L-proline, and L-methionine were significantly reduced. L-methionine is an essential amino acid, and Roediger et al. found methionine has a protective effect on sulfide-induced acute oxidative damage in rat colon cells [40]. L-aspartic acid is the synthetic precursor of L-methionine in the body and can be combined with various amino acids to make active drugs such as fatigue recovery agents [41–43]. In addition, Notararigo et al. reported that the levels of L-proline and L-methionine in UC patients were significantly lower than those in healthy controls, which were related to the impaired intestinal mucosal barrier, intestinal microbiota disturbance, and impaired absorption capacity in UC patients [44]. Consistent with the above studies, we showed that the rats in the DSS model group were emaciated, lethargic, and significantly decreased in activity, which may be related to the lower levels of L-aspartic acid and L-methionine, reflecting the insufficient intestinal energy absorption including essential amino acids [38, 45]. Moreover, differential metabolites were mainly involved in alanine, aspartate, and glutamate metabolism. Hong et al. reported that compound sophorae decoction can significantly improve the symptoms of DSS-induced colitis by modulating alanine, aspartate, and glutamate metabolism [46]. Therefore, artemisinin might alleviate the inflammation in UC by alanine, aspartate, and glutamate metabolism.

Creatinine and L-carnitine are involved in the energy supply of mammalian cells [47]. Creatine is an important compound for energy storage and utilization [48], and creatinine is the breakdown product of creatine phosphate in muscles [47]. In this study, creatinine was significantly increased in the MG, indicating that DSS-induced colitis rats have a disordered energy supply. L-carnitine is involved in the metabolism of most mammals, plants, and some bacteria and plays a key role in lipid metabolism and *β*-oxidation, and it is used to transport long-chain fatty acids into mitochondria to be oxidized for energy production and is an important part of muscle energy metabolism [49]. Studies have reported that under the influence of oxidative stress and weight loss in DSS-induced colitis, carnitine and creatine typically increase during disease development, which may reflect the overall stress state of the animals and further indicates that ATP and fatty acids are required for energy supply during UC [50, 51]. However, this situation was reversed after artemisinin intervention, indicating that artemisinin has positive effects on regulating stress response and energy supply in UC rats.

Synthesis and degradation of ketone bodies are other important pathways for artemisinin to interfere with the occurrence and development of UC. A previous study reported that diet-induced ketolytic metabolism can significantly reduce pain and inflammation [52]. Another study demonstrated that increased ketogenesis can mitigate TNF*α*-induced intestinal cells apoptosis and inflammation, suggesting that ketogenesis has a protective role in TNF*α*-induced intestinal pathology [53]. In this study, we showed that artemisinin could alleviate the inflammatory response in UC rats, indicating that artemisinin may play a role in regulating the synthesis and degradation of ketone bodies in UC rats.

In the present study, the expressions of glucose and gluconic acid were upregulated in MG rats, suggesting that DSS-induced colitis rats had an obvious disorder of glucose metabolism, such as decreased glucose decomposition, enhanced gluconeogenesis, and weakened glycolysis. It was reported that UC patients with high endoscopic activity [UCEIS ≥ 3] had significantly elevated glucose concentration [38], and high glucose levels were also found in fecal extracts and other biological specimens of UC patients [54]. This indicates that energy-related metabolites in the colon play an important role in maintaining the balance of gut microbiota and intestinal cells [55, 56], and the elevated glucose levels in DSS model rats may be caused by the energy disturbance of the host-microbiome system [57, 58]. Nevertheless, glucose and gluconic acid decreased after artemisinin intervention, demonstrating that the glycolysis pathway and energy metabolism disorders in rats were improved.

## 5. Conclusion

In this study, we performed a bioinformatics approach to identify DEGs in UC. In order to study the protective effect of artemisinin on DSS-induced colitis, we conducted animal experiments to verify the IL-17 signaling pathway, which is mainly enriched in UC DEGs and preliminarily explored the changes of endogenous metabolites in serum of UC rats after artemisinin intervention. These results demonstrate that artemisinin can regulate the balance of proinflammatory and anti-inflammatory factors, degrade the inflammatory response of UC, and adjust amino acid metabolism and synthesis and degradation of ketone bodies, which are related to energy metabolism and antioxidant capacity. This study aimed to provide a potential new strategy for the treatment of UC.

## Figures and Tables

**Table 1:** The standard of DAI score.

Score	Decline in body weight (%)	Stool	Hematochezia
0	Naught	Normal	Negative
1	1–5	—	—
2	5–10	Semi-loose	BLD (+)
3	10–15	—	—
4	>15	Loose	Bloody eye

**Table 2:** Statistical analysis of potential metabolites of ulcerative colitis rats intervened by artemisinin.

No.	Diﬀerential metabolites	Formula	MG vs NG		AG vs MG	
			*p* value	Up/down	*p* value	Up/down
1	(R)-3-hydroxybutyric acid	C4H8O3	0.0067284	Down	0.023948675	Up
2	Jasmonic acid	C12H18O3	0.001182325	Down	0.007405533	Up
3	Homovanillin	C9H10O3	0.045589156	Down	0.000939106	Up
4	Gentisic acid	C7H6O4	0.002915086	Down	0.001947528	Up
5	Pyrimidodiazepine	C9H11N5O2	0.000862359	Down	0.001947528	Up
6	Cysteine-S-sulfate	C3H7NO5S2	0.036795147	Down	0.003876041	Up
7	1H-indole-3-acetamide	C10H10N2O	0.002915086	Down	0.010081694	Up
8	14,15-DiHETrE	C20H34O4	0.008763529	Down	0.007405533	Up
9	5-methylcytosine	C5H7N3O	0.000448668	Down	0.040568856	Up
10	p-aminobenzoic acid	C7H7NO2	0.036795147	Down	0.031324131	Up
11	Maleic acid	C4H4O4	0.045589156	Down	0.031324131	Up
12	L-aspartic acid	C4H7NO4	0.002915086	Down	0.040568856	Up
13	3-methylthiopropionic acid	C4H8O2S	0.011331976	Down	0.018129008	Up
14	L-proline	C5H9NO2	0.014548024	Down	0.040568856	Up
15	Dimethyl sulfone	C2H6O2S	0.018543313	Down	0.001947528	Up
16	L-methionine	C5H11NO2S	0.023467646	Down	0.00538494	Up
17	Pyrrolidonecarboxylic acid	C5H7NO3	0.000624338	Up	0.031324131	Down
18	L-malic acid	C4H6O5	0.018543313	Up	0.000939106	Down
19	L-carnitine	C7H16NO3	0.002173751	Up	0.023948675	Down
20	Isocitric acid	C6H8O7	0.003880688	Up	0.002761604	Down
21	Indoleglycerol phosphate	C11H14NO6P	0.000862359	Up	0.013587273	Down
22	Dethiobiotin	C10H18N2O3	0.045589156	Up	0.000939106	Down
23	S-lactoylglutathione	C13H21N3O8S	0.014548024	Up	0.002761604	Down
24	3-(3,4-dihydroxyphenyl)pyruvate	C9H7O5	0.008763529	Up	0.031324131	Down
25	Gluconic acid	C6H12O7	0.00512852	Up	0.00538494	Down
26	Phenylacetic acid	C8H8O2	0.001609069	Up	0.001359376	Down
27	Creatinine	C4H7N3O	0.023467646	Up	0.040568856	Down
28	D-glucose	C6H12O6	0.018543313	Up	0.000409933	Down
29	Thymine	C5H6N2O2	0.018543313	Up	0.000409933	Down
30	O-phosphoethanolamine	C2H8NO4P	0.001182325	Up	0.001359376	Down
31	Deoxyuridine	C9H12N2O5	0.0067284	Up	0.007405533	Down
32	Pimelic acid	C7H12O4	0.00512852	Up	0.001947528	Down
33	6-ketoprostaglandin E1	C20H32O6	0.011331976	Up	0.013587273	Down

## Data Availability

The datasets used and/or analyzed during the current study are available from the corresponding author on reasonable request.

## Supplementary Materials

Supplementary Materials Table S1: the overlapping differential genes in two databases (GSE36807 and GSE9452) in ulcerative colitis. Table S2: enriched terms in the KEGG pathways for DEGs in ulcerative colitis (top 20). Table S3: main metabolic pathways information of impact >0.1.

## References

[1] Ma C., Sedano R., Almradi A. (2021). An international consensus to standardize integration of histopathology in ulcerative colitis clinical trials. *Gastroenterology*.

[2] Dal Buono A., Roda G., Argollo M., Paridaens K., Peyrin-Biroulet L., Danese S. (2021). Treat to target’ in mild to moderate ulcerative colitis: evidence to support this strategy. *Current Drug Targets*.

[3] Wei S.-C., Sollano J., Hui Y. T. (2021). Epidemiology, burden of disease, and unmet needs in the treatment of ulcerative colitis in Asia. *Expert Review of Gastroenterology & Hepatology*.

[4] Ishida N., Onoue S., Miyazu T. (2021). Further research on the clinical relevance of the ulcerative colitis colonoscopic index of severity for predicting 5-year relapse. *International Journal of Colorectal Disease*.

[5] Rabbenou W., Ullman T. A. (2020). Risk of colon cancer and recommended surveillance strategies in patients with ulcerative colitis. *Gastroenterology Clinics of North America*.

[6] Zhang J., Wang X., Xu L., Zhang Z., Wang F., Tang X. (2020). Investigation of potential genetic biomarkers and molecular mechanism of ulcerative colitis utilizing bioinformatics analysis. *BioMed Research International*.

[7] Hu Z. R., Zheng W. J., Yan Q., Hu W. W., Sun X. S. (2020). [Bioinformatic analysis of differentially expressed genes and Chinese medicine prediction for ulcerative colitis]. *Zhongguo Zhongyao Zazhi*.

[8] Wang D., Ma X., Guo S. (2020). Effect of huangqin tang on urine metabolic profile in rats with ulcerative colitis based on UPLC-Q-exactive orbitrap MS. *Evidence-based Complementary and Alternative Medicine: eCAM*.

[9] Ma N., Zhang Z., Liao F., Jiang T., Tu Y. (2020). The birth of artemisinin. *Pharmacology & Therapeutics*.

[10] Kiani B. H., Kayani W. K., Khayam A. U., Dilshad E., Ismail H., Mirza B. (2020). Artemisinin and its derivatives: a promising cancer therapy. *Molecular Biology Reports*.

[11] Li T., Chen H., Wei N. (2012). Anti-inflammatory and immunomodulatory mechanisms of artemisinin on contact hypersensitivity. *International Immunopharmacology*.

[12] Kim H. G., Yang J. H., Han E. H. (2013). Inhibitory effect of dihydroartemisinin against phorbol ester-induced cyclooxygenase-2 expression in macrophages. *Food and Chemical Toxicology*.

[13] Lee A. S., Hur H. J., Sung M. J. (2020). The effect of artemisinin on inflammation-associated lymphangiogenesis in experimental acute colitis. *International Journal of Molecular Sciences*.

[14] Hu D., Wang Y., Chen Z. (2014). Artemisinin protects against dextran sulfate-sodium-induced inflammatory bowel disease, which is associated with activation of the pregnane X receptor. *European Journal of Pharmacology*.

[15] Liu F., Yao Y., Lu Z. (2021). 5-Hydroxy-4-methoxycanthin-6-one alleviates dextran sodium sulfate-induced colitis in rats via regulation of metabolic profiling and suppression of NF-*κ*B/p65 signaling pathway. *Phytomedicine*.

[16] Psychogios N., Hau D. D., Peng J. (2011). The human serum metabolome. *PLoS One*.

[17] Zhao H., Liu Y., Li Z. (2018). Identification of essential hypertension biomarkers in human urine by non-targeted metabolomics based on UPLC-Q-TOF/MS. *Clinica Chimica Acta*.

[18] Chu H., Zhang A., Han Y. (2016). Metabolomics approach to explore the effects of Kai-Xin-San on Alzheimer’s disease using UPLC/ESI-Q-TOF mass spectrometry. *Journal of Chromatography B*.

[19] Fritsch J., Garces L., Quintero M. A. (2021). Low-fat, high-fiber diet reduces markers of inflammation and dysbiosis and improves quality of life in patients with ulcerative colitis. *Clinical Gastroenterology and Hepatology*.

[20] Murano M., Maemura K., Hirata I. (2000). Therapeutic effect of intracolonically administered nuclear factor kappa B (p65) antisense oligonucleotide on mouse dextran sulphate sodium (DSS)-induced colitis. *Clinical and Experimental Immunology*.

[21] Matson J., Ramamoorthy S., Lopez N. E. (2021). The role of biomarkers in surgery for ulcerative colitis: a review. *Journal of Clinical Medicine*.

[22] Zhu F., Ke Y., Luo Y. (2021). Effects of different treatment of fecal microbiota transplantation techniques on treatment of ulcerative colitis in rats. *Frontiers in Microbiology*.

[23] Arafat E. A., Marzouk R. E., Mostafa S. A., Hamed W. H. E. (2021). Identification of the molecular basis of nanocurcumin-induced telocyte preservation within the colon of ulcerative colitis rat model. *Mediators of Inflammation*.

[24] Shi L., Han X., Li J.-X. (2020). Identification of differentially expressed genes in ulcerative colitis and verification in a colitis mouse model by bioinformatics analyses. *World Journal of Gastroenterology*.

[25] Clough E., Barrett T. (2016). The gene expression Omnibus database. *Methods in Molecular Biology*.

[26] Zhu Q., Zheng P., Chen X., Zhou F., He Q., Yang Y. (2018). Andrographolide presents therapeutic effect on ulcerative colitis through the inhibition of IL-23/IL-17 axis. *American Journal of Tourism Research*.

[27] Zhang X., Deng Q.-H., Deng J.-H., Wang S.-J., Chen Q. (2020). Lovastatin derivative dehydrolovastatin ameliorates ulcerative colitis in mice by suppressing NF-*κ*B and inflammatory cytokine expression. *Korean Journal of Physiology and Pharmacology*.

[28] Ding Y., Chen M., Wang Q. (2020). Integrating pharmacology and microbial network analysis with experimental validation to reveal the mechanism of composite Sophora colon-soluble capsule against ulcerative colitis. *Evidence-based Complementary and Alternative Medicine: eCAM*.

[29] Niu K., Li Q., Liu Y. (2021). Molecular targets and mechanisms of scutellariae radix-coptidis rhizoma drug pair for the treatment of ulcerative colitis based on network pharmacology and molecular docking. *Evidence Based Complement Alternative Medicine*.

[30] Pushparaj P. N., Li D., Komai-Koma M. (2013). Interleukin-33 exacerbates acute colitis via interleukin-4 in mice. *Immunology*.

[31] Ng S. C., Kamm M. A. (2008). Review article: new drug formulations, chemical entities and therapeutic approaches for the management of ulcerative colitis. *Alimentary Pharmacology & Therapeutics*.

[32] Liu Y., Qu Y., Liu L. (2019). PPAR-*γ* agonist pioglitazone protects against IL-17 induced intervertebral disc inflammation and degeneration via suppression of NF-*κ*B signaling pathway. *International Immunopharmacology*.

[33] Kanazawa T., Taneike I., Akaishi R. (2004). Amino acids and insulin control autophagic proteolysis through different signaling pathways in relation to mTOR in isolated rat hepatocytes. *Journal of Biological Chemistry*.

[34] Scott S. A., Fu J., Chang P. V. (2020). Microbial tryptophan metabolites regulate gut barrier function via the aryl hydrocarbon receptor. *Proceedings of the National Academy of Sciences*.

[35] Nikolaus S., Schulte B., Al-Massad N. (2017). Increased tryptophan metabolism is associated with activity of inflammatory bowel diseases. *Gastroenterology*.

[36] Brencher L., Petrat F., Stych K., Hamburger T., Kirsch M. (2017). Effect of Glycine, pyruvate, and resveratrol on the regeneration process of postischemic intestinal mucosa. *BioMed Research International*.

[37] Giris M., Depboylu B., Dogru-Abbasoglu S. (2008). Effect of taurine on oxidative stress and apoptosis-related protein expression in trinitrobenzene sulphonic acid-induced colitis. *Clinical and Experimental Immunology*.

[38] Probert F., Walsh A., Jagielowicz M. (2018). Plasma nuclear magnetic resonance metabolomics discriminates between high and low endoscopic activity and predicts progression in a prospective cohort of patients with ulcerative colitis. *Journal of Crohn’s and Colitis*.

[39] Sakata T., Hana K., Mikami T., Yoshida T., Endou H., Okayasu I. (2020). Positive correlation of expression of L-type amino-acid transporter 1 with colorectal tumor progression and prognosis: higher expression in sporadic colorectal tumors compared with ulcerative colitis-associated neoplasia. *Pathology, Research & Practice*.

[40] Roediger W. E., Babidge W., Millard S. (1996). Methionine derivatives diminish sulphide damage to colonocytes--implications for ulcerative colitis. *Gut*.

[41] Benveniste H., Drejer J., Schousboe A., Diemer N. H. (1984). Elevation of the extracellular concentrations of glutamate and aspartate in rat hippocampus during transient cerebral ischemia monitored by intracerebral microdialysis. *Journal of Neurochemistry*.

[42] Xiao G., Yang J., Yan L. (2015). Comparison of diagnostic accuracy of aspartate aminotransferase to platelet ratio index and fibrosis-4 index for detecting liver fibrosis in adult patients with chronic hepatitis B virus infection: a systemic review and meta-analysis. *Hepatology*.

[43] Liu F., Patterson T. A., Sadovova N. (2013). Ketamine-induced neuronal damage and altered N-methyl-D-aspartate receptor function in rat primary forebrain culture. *Toxicological Sciences*.

[44] Notararigo S., Martín-Pastor M., Viñuela-Roldán J. E., Quiroga A., Dominguez-Munoz J. E., Barreiro-de Acosta M. (2021). Targeted 1H NMR metabolomics and immunological phenotyping of human fresh blood and serum samples discriminate between healthy individuals and inflammatory bowel disease patients treated with anti-TNF. *Journal of Molecular Medicine*.

[45] Schicho R., Shaykhutdinov R., Ngo J. (2012). Quantitative metabolomic profiling of serum, plasma, and urine by 1H NMR spectroscopy discriminates between patients with inflammatory bowel disease and healthy individuals. *Journal of Proteome Research*.

[46] Hong Z. C, Cai Q., Duan X. Y. (2021). Effect of compound Sophorae decoction in the treatment of ulcerative colitis by tissue extract metabolomics approach. *Journal of traditional Chinese medicine = Chung i tsa chih ying wen pan*.

[47] Izquierdo-Garcia J. L., Comella-Del-Barrio P., Campos-Olivas R. (2020). Discovery and validation of an NMR-based metabolomic profile in urine as TB biomarker. *Scientific Reports*.

[48] Colas C., Banci G., Martini R., Ecker G. F. (2020). Studies of structural determinants of substrate binding in the Creatine Transporter (CreaT, SLC6A8) using molecular models. *Scientific Reports*.

[49] Zhang H., Fu P., Ke B. (2014). Metabolomic analysis of biochemical changes in the plasma and urine of collagen-induced arthritis in rats after treatment with Huang-Lian-Jie-Du-Tang. *Journal of Ethnopharmacology*.

[50] Lee R., West D., Phillips S. M., Britz-McKibbin P. (2010). Differential metabolomics for quantitative assessment of oxidative stress with strenuous exercise and nutritional intervention: thiol-specific regulation of cellular metabolism with N-acetyl-L-cysteine pretreatment. *Analytical Chemistry*.

[51] Connor S. C., Wu W., Sweatman B. C. (2004). Effects of feeding and body weight loss on the1H-NMR-based urine metabolic profiles of male Wistar Han Rats: implications for biomarker discovery. *Biomarkers*.

[52] Ruskin D. N., Kawamura M., Masino S. A. (2009). Reduced pain and inflammation in juvenile and adult rats fed a ketogenic diet. *PLoS One*.

[53] Kim J. T., Napier D. L., Kim J. (2021). Ketogenesis alleviates TNF*α*-induced apoptosis and inflammatory responses in intestinal cells. *Free Radical Biology and Medicine*.

[54] Le Gall G., Noor S. O., Ridgway K. (2011). Metabolomics of fecal extracts detects altered metabolic activity of gut microbiota in ulcerative colitis and irritable bowel syndrome. *Journal of Proteome Research*.

[55] Lin X., Liu X., Xu J. (2019). Metabolomics analysis of herb-partitioned moxibustion treatment on rats with diarrhea-predominant irritable bowel syndrome. *Chinese Medicine*.

[56] Jacobs S. R., Herman C. E., Maciver N. J. (2008). Glucose uptake is limiting in T cell activation and requires CD28-mediated Akt-dependent and independent pathways. *The Journal of Immunology*.

[57] Abdallah H. M. I., Ammar N. M., Abdelhameed M. F. (2020). Protective mechanism of Acacia saligna butanol extract and its nano-formulations against ulcerative colitis in rats as revealed via biochemical and metabolomic assays. *Biology*.

[58] Farag M. A., Abdelwareth A., Sallam I. E. (2020). Metabolomics reveals impact of seven functional foods on metabolic pathways in a gut microbiota model. *Journal of Advanced Research*.

