# Supplementary material for: Artemisinin Alleviates Intestinal Inflammation and Metabolic Disturbance in Ulcerative Colitis Rats Induced by DSS
Source: Evid Based Complement Alternat Med. 2022 Apr 19;2022:6211215. doi: 10.1155/2022/6211215 (PMC9042626; doi:10.1155/2022/6211215)
Supplement: Supplementary Materials — Table S1: the overlapping differential genes in two databases (GSE36807 and GSE9452) in ulcerative colitis. Table S2: enriched terms in the KEGG pathways for DEGs in ulcerative colitis (top 20). Table S3: main metabolic pathways information of impact >0.1. [file 6211215.f1.zip › 6211215.f1/Table S1.docx]

**Table S1 The overlapping differential genes in two databases (GSE36807 and GSE9452) in ulcerative colitis.**

| DEGs | Gene Symbol |
| --- | --- |
| Up-regulated genes (32) | GHR、PCK1、CDHR1、HMGCS2、CHP2、GUCA2A、ABCB1、CKB 、ADH1C、  NPY1R、CCL23、SATB2-AS1、C21orf88、ZNF415、MCOLN2、BCHE、MT1H、  KCNJ2、KIAA0895L、ENTPD5、LRRN2、VLDLR、AKAP7、HSD11B2、VIPR1、  STX19、TSPAN7、IL1R2、VSTM2A、PRDX6、CA2、CPA6 |
| Down-regulated genes (138) | ELMO1、ICAM1、IFITM2、STAT1、CD40、NR2F1、LAG3、ABCC1、ST3GAL1、SLAMF6、CDC45、IL2RA、TRIB2、WARS、PCOLCE、RHOH、EGR3、HLA-DPB1、IFNG、VSNL1、SERPINE1、GBP1、TRIM40、HSD11B1、P2RY6、GJA4、ADA、CD86、RAC2、ST3GAL5、OAS2、ANXA1、FCN3、COL1A2、MICB、FGR、APOL1、STS、FCGR2C、SLC7A11、SELP、SLA、UBE2L6、ANKRD36BP2、TLR2、ITGA4、THEMIS2、C1S、BCL2A1、SPI1、SCD、ANLN、CTSK、CBR3、LUM、FCN1、COL6A3、GNA15、BTN3A3、WNT5A、THY1、IFIT3、SLC7A5、LPCAT1、TGFBI、KIAA0125、PECAM1、PLAU、HCLS1、APOBEC1、SLC43A3、DDIT4、ITGB2、KYNU、CASP1、CXCL11、SERPINA3、PNOC、CSF3R、RARRES3、CCR1、CASP5、CDH3、IL1B、CD163、ENTPD1、S100P、PDZK1IP1、PSMB9、LILRA6、CFI、C4BPB、MNDA、FUT8、HCAR3、DMBT1、CXCL6、AGT、C4BPA、CSTA、NCF2、TIMP1、MMP10、PCSK1、AZGP1、CXCL3、VNN1、CXCR2、CD38、AQP9、IGHM、GZMB、LAX1、CCL4、CXCL9、CHI3L1、SPINK4、REG3A、NOS2、S100A8、FCGR3B、CCL11、IDO1、DEFA6、TCN1、  MMP12、MMP1、CXCL8、LY96、MMP7、CXCL10、PI3、MMP3、CXCL1、S100A9、DEFA5、REG1B、REG1A |
