# Supplementary material for: Artemisinin Alleviates Intestinal Inflammation and Metabolic Disturbance in Ulcerative Colitis Rats Induced by DSS
Source: Evid Based Complement Alternat Med. 2022 Apr 19;2022:6211215. doi: 10.1155/2022/6211215 (PMC9042626; doi:10.1155/2022/6211215)
Supplement: Supplementary Materials — Table S1: the overlapping differential genes in two databases (GSE36807 and GSE9452) in ulcerative colitis. Table S2: enriched terms in the KEGG pathways for DEGs in ulcerative colitis (top 20). Table S3: main metabolic pathways information of impact >0.1. [file 6211215.f1.zip › 6211215.f1/Table S2.docx]

| ID | Description | GeneRatio | BgRatio | pvalue | p.adjust | qvalue | Count |
| --- | --- | --- | --- | --- | --- | --- | --- |
| hsa05323 | Rheumatoid arthritis | 14/114 | 93/8086 | 0.0000000000319 | 0.00000000653 | 0.00000000503 | 14 |
| hsa04061 | Viral protein interaction with cytokine and cytokine receptor | 13/114 | 100/8086 | 0.00000000111 | 0.000000113 | 0.0000000874 | 13 |
| hsa04657 | IL-17 signaling pathway | 12/114 | 94/8086 | 0.00000000622 | 0.000000425 | 0.000000327 | 12 |
| hsa04062 | Chemokine signaling pathway | 16/114 | 192/8086 | 0.00000000917 | 0.00000047 | 0.000000362 | 16 |
| hsa04620 | Toll-like receptor signaling pathway | 12/114 | 104/8086 | 0.00000002 | 0.000000724 | 0.000000558 | 12 |
| hsa04060 | Cytokine-cytokine receptor interaction | 19/114 | 295/8086 | 0.0000000236 | 0.000000724 | 0.000000558 | 19 |
| hsa05144 | Malaria | 9/114 | 50/8086 | 0.0000000247 | 0.000000724 | 0.000000558 | 9 |
| hsa05133 | Pertussis | 10/114 | 76/8086 | 0.0000000912 | 0.00000234 | 0.0000018 | 10 |
| hsa05140 | Leishmaniasis | 10/114 | 77/8086 | 0.000000104 | 0.00000236 | 0.00000182 | 10 |
| hsa05146 | Amoebiasis | 11/114 | 102/8086 | 0.000000163 | 0.00000335 | 0.00000258 | 11 |
| hsa05417 | Lipid and atherosclerosis | 14/114 | 215/8086 | 0.00000173 | 0.0000306 | 0.0000236 | 14 |
| hsa04064 | NF-kappa B signaling pathway | 10/114 | 104/8086 | 0.00000179 | 0.0000306 | 0.0000236 | 10 |
| hsa05202 | Transcriptional misregulation in cancer | 13/114 | 192/8086 | 0.0000027 | 0.0000425 | 0.0000327 | 13 |
| hsa05150 | Staphylococcus aureus infection | 9/114 | 96/8086 | 0.00000745 | 0.000109098 | 0.000084 | 9 |
| hsa05134 | Legionellosis | 7/114 | 57/8086 | 0.0000136 | 0.00018581 | 0.000143114 | 7 |
| hsa04621 | NOD-like receptor signaling pathway | 11/114 | 186/8086 | 0.0000582 | 0.000745102 | 0.000573891 | 11 |
| hsa05143 | African trypanosomiasis | 5/114 | 37/8086 | 0.000155107 | 0.001870407 | 0.001440622 | 5 |
| hsa05330 | Allograft rejection | 5/114 | 38/8086 | 0.000176615 | 0.001893365 | 0.001458304 | 5 |
| hsa04610 | Complement and coagulation cascades | 7/114 | 85/8086 | 0.000183491 | 0.001893365 | 0.001458304 | 7 |
| hsa05416 | Viral myocarditis | 6/114 | 60/8086 | 0.000185386 | 0.001893365 | 0.001458304 | 6 |

**Table S2 Enriched terms in the KEGG pathways for DEGs in ulcerative colitis (top 20).**
