# Supplementary material for: Artemisinin Alleviates Intestinal Inflammation and Metabolic Disturbance in Ulcerative Colitis Rats Induced by DSS
Source: Evid Based Complement Alternat Med. 2022 Apr 19;2022:6211215. doi: 10.1155/2022/6211215 (PMC9042626; doi:10.1155/2022/6211215)
Supplement: Supplementary Materials — Table S1: the overlapping differential genes in two databases (GSE36807 and GSE9452) in ulcerative colitis. Table S2: enriched terms in the KEGG pathways for DEGs in ulcerative colitis (top 20). Table S3: main metabolic pathways information of impact >0.1. [file 6211215.f1.zip › 6211215.f1/Table S3.docx]

**Table S3 Main metabolic pathways information of impact > 0.1.**

| Metabolic pathway | Raw p | -LOG(p) | Impact | Compounds |
| --- | --- | --- | --- | --- |
| Renal cell carcinoma | 0.029577 | 3.5208 | 0.4 | L-Malic acid |
| Synthesis and degradation of ketone bodies | 0.058305 | 2.8421 | 0.13636 | (R)-3-Hydroxybutyric acid |
| Alanine, aspartate and glutamate metabolism | 0.24519 | 1.4057 | 0.15169 | L-Aspartate |
| Central carbon metabolism in cancer | 0.0000248 | 10.605 | 0.11321 | Aspartate, L-Methionine, L-Proline, L-Malic acid, (S)-Malate, Isocitrate |
